# Differences in microRNA-29 and Pro-fibrotic Gene Expression in Mouse and Human Hypertrophic Cardiomyopathy

**DOI:** 10.3389/fcvm.2019.00170

**Published:** 2019-12-17

**Authors:** Yamin Liu, Junaid Afzal, Styliani Vakrou, Gabriela V. Greenland, C. Conover Talbot, Virginia B. Hebl, Yufan Guan, Rehan Karmali, Jil C. Tardiff, Leslie A. Leinwand, Jeffrey E. Olgin, Samarjit Das, Ryuya Fukunaga, M. Roselle Abraham

**Affiliations:** ^1^Division of Cardiology, Hypertrophic Cardiomyopathy Center of Excellence, University of California, San Francisco, San Francisco, CA, United States; ^2^Hypertrophic Cardiomyopathy Center of Excellence, Johns Hopkins University, Baltimore, MD, United States; ^3^Johns Hopkins School of Medicine, Institute for Basic Biomedical Sciences, Baltimore, MD, United States; ^4^Intermountain Medical Center, Intermountain Heart Institute, Murray, UT, United States; ^5^Sarver Heart Center, University of Arizona Health Sciences, Tucson, AZ, United States; ^6^Molecular, Cellular and Developmental Biology, Biofrontiers Institute, University of Colorado, Boulder, CO, United States; ^7^Department of Anesthesia and Critical Care Medicine, Johns Hopkins University, Baltimore, MD, United States; ^8^Department of Biological Chemistry, Johns Hopkins School of Medicine, Baltimore, MD, United States

**Keywords:** hypertrophic cardiomyopathy, miR-29, TGF-beta, collagen, mouse, humans

## Abstract

**Background:** Hypertrophic cardiomyopathy (HCM) is characterized by myocyte hypertrophy and fibrosis. Studies in two mouse models (R92W-TnT/R403Q-MyHC) at early HCM stage revealed upregulation of endothelin (ET1) signaling in both mutants, but TGFβ signaling only in TnT mutants. Dysregulation of miR-29 expression has been implicated in cardiac fibrosis. But it is unknown whether expression of miR-29a/b/c and profibrotic genes is commonly regulated in mouse and human HCM.

**Methods:** In order to understand mechanisms underlying fibrosis in HCM, and examine similarities/differences in expression of miR-29a/b/c and several profibrotic genes in mouse and human HCM, we performed parallel studies in rat cardiac myocyte/fibroblast cultures, examined gene expression in two mouse models of (*non-obstructive*) HCM (R92W-TnT, R403Q-MyHC)/controls at early (5 weeks) and established (24 weeks) disease stage, and analyzed publicly available mRNA/miRNA expression data from *obstructive*-HCM patients undergoing septal myectomy/controls (unused donor hearts).

**Results:** Myocyte cultures: ET1 increased superoxide/H_2_O_2_, stimulated TGFβ expression/secretion, and suppressed miR-29a expression in myocytes. The effect of ET1 on miR-29 and TGFβ expression/secretion was antagonized by N-acetyl-cysteine, a reactive oxygen species scavenger. Fibroblast cultures: ET1 had no effect on pro-fibrotic gene expression in fibroblasts. TGFβ1/TGFβ2 suppressed miR-29a and increased collagen expression, which was abolished by miR-29a overexpression. Mouse and human HCM: Expression of miR-29a/b/c was lower, and *TGFB1*/collagen gene expression was higher in TnT mutant-LV at 5 and 24 weeks; no difference was observed in expression of these genes in MyHC mutant-LV and in human myectomy tissue. *TGFB2* expression was higher in LV of both mutant mice and human myectomy tissue. *ACE2*, a negative regulator of the renin-angiotensin-aldosterone system, was the most upregulated transcript in human myectomy tissue. Pathway analysis predicted upregulation of the anti-hypertrophic/anti-fibrotic liver X receptor/retinoid X receptor (LXR/RXR) pathway only in human myectomy tissue.

**Conclusions:** Our *in vitro* studies suggest that activation of ET1 signaling in cardiac myocytes increases reactive oxygen species and stimulates TGFβ secretion, which downregulates miR-29a and increases collagen in fibroblasts, thus contributing to fibrosis. Our gene expression studies in mouse and human HCM reveal allele-specific differences in miR-29 family/profibrotic gene expression in mouse HCM, and activation of anti-hypertrophic/anti-fibrotic genes and pathways in human HCM.

## Introduction

Hypertrophic cardiomyopathy (HCM), most frequently caused by mutations in sarcomeric protein genes, manifests as myocyte hypertrophy, myocyte disarray, fibrosis, and arteriolar remodeling (1). Expression of the pathologic hallmarks of HCM vary with disease stage and underlying mutation. Our studies comparing two mouse models of HCM (R92W-TnT, R403Q-MyHC) with mutations in cardiac troponin T (cTnT) (2, 3) and myosin heavy chain (MyHC) genes (4, 5), at an early stage of disease (5 weeks of age), revealed differences in expression of *TGFB1/3*, microRNA-29 (miR-29), collagen genes, and redox environment in the two mutant mouse lines (6). Dysregulation of cardiac miR-29 family (miR-29a/b/c) expression has been implicated in cardiac fibrosis in experimental models (7), and circulating miR-29a has been associated with cardiac fibrosis in HCM patients (8). But it is unknown whether miR-29 dysregulation varies with disease stage in HCM mouse models, and whether cardiac miR-29 expression is changed in human HCM (8). In order to address this question, we examined expression of *TGFB1-3*, miR-29a/b/c, and collagen genes in the left ventricle (LV) and left atrium (LA) of TnT/MyHC mutants and littermate control mice at 5 and 24 weeks of age, and analyzed publicly available gene (mRNA, miRNA) expression data from human myectomy samples and control subjects.

In our recently published study, Ingenuity Pathway analysis (IPA) of gene (mRNA) expression data in 5 weeks old mouse hearts (R92W-TnT, R403Q-MyHC and littermate controls) predicted upregulation of endothelin1 (ET1) and transforming growth factor-beta signaling only in TnT mutants, that demonstrated an oxidized redox environment (6). Prior studies have demonstrated upregulation of TGFβ by mitochondrial reactive oxygen species (ROS) in lung fibroblasts (9), and by ET1 in cardiac myocytes (10). Additionally, ROS has been implicated as a mediator of ET1-induced cardiac hypertrophy (11, 12). We hypothesized that activation of ET1 signaling in cardiac myocytes increases ROS levels, which stimulates TGFβ secretion by cardiac myocytes, and collagen expression in cardiac fibroblasts. We tested this hypothesis by assessing miR-29 and select pro-fibrotic gene expression in (1) cardiac myocytes/fibroblasts following ET1 treatment, and (2) in cardiac fibroblasts following ET1, TGFβ1, and TGFβ2 treatment. Our results indicate that ET1 increases ROS and stimulates TGFβ secretion by cardiac myocytes, which suppresses miR-29 and increases collagen expression in cardiac fibroblasts.

## Materials and Methods

### Study Design

**Figure 1:**
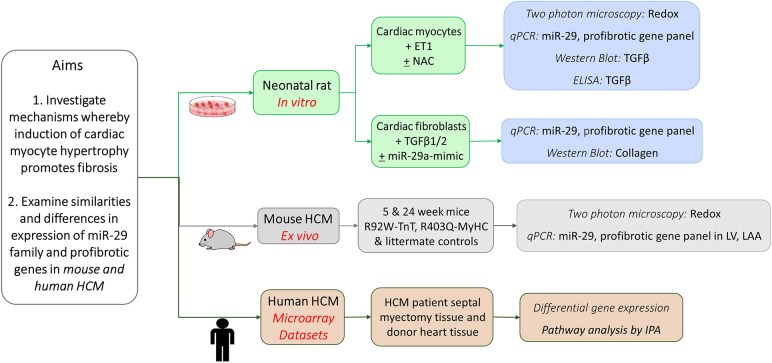
Schematic for overall study design.

We performed studies in cardiac myocytes and cardiac fibroblasts derived from neonatal rat hearts, examined redox/gene expression in two mouse models of HCM/littermate controls at early and established disease stage, and analyzed publicly available gene expression data from HCM patients undergoing myectomy and controls, with a focus on TGFβ, miR-29 family, and its profibrotic target genes (Figure 1).

Studies in cardiac myocytes and fibroblasts obtained from healthy neonatal rat hearts were used to examine the effect of ET1 and redox modulation of cardiac myocytes on TGFβ signaling in cardiac myocytes, and collagen expression in cardiac fibroblasts. We examined miR-29a/b/c and profibrotic gene expression in the left ventricle (LV) and left atrial appendage (LA appendage) in 2 HCM mouse models because of the high incidence of cardiac fibrosis and ventricular/atrial arrhythmias in HCM patients. These two mouse models of sarcomeric HCM (R92W-TnT, R403Q-MyHC) are based on mutations that lead to sudden cardiac death at a young age in the setting of fibrosis/mild hypertrophy (13, 14), and significant LV hypertrophy/fibrosis/heart failure requiring heart transplantation in middle age (15, 16). Since obtaining endomyocardial biopsies from HCM patients for research is not feasible, we analyzed publicly available microarray datasets from HCM patients undergoing myectomy (compared to tissue obtained from unused donor hearts/controls) to assess whether expression of TGFB1-3, miR-29a/b/c, and its profibrotic target genes is dysregulated in human HCM.

### Myocyte and Fibroblast Isolation From Neonatal Rat Hearts

Cardiac myocytes and cardiac fibroblasts were isolated from neonatal rat hearts as described previously (17). Briefly hearts from newborn Sprague Dawley rats (2–3 days old) were quickly excised, minced and digested with trypsin (at 4°C for 16–20 h) followed by collagenase type 2 (37°C for 30–45 min), following which cell suspensions were centrifuged at 100× g for 5 min. The pellet was re-suspended in Dulbecco's Modified Eagle's Medium (DMEM) containing 4.5 g/L D-glucose, 10% fetal bovine serum (FBS), 100 U/mL penicillin/streptomycin (Pen/Strep), and transferred to a flask for 1 h for preferential attachment of fibroblasts. After 1 h, cardiac myocytes were plated in six well plates, at a density of 6 × 10^5^ per well. Fibroblasts adherent to the flask were cultured in DMEM containing 4.5 g/L D-glucose, 10% FBS, 100 U/mL Pen/Strep for the first 24 h. Fibroblasts were expanded in DMEM containing 1 gm/L D-glucose, 10% FBS, 100 U/mL Pen/Strep. Fibroblasts were allowed to proliferate until they were 70% confluent; fibroblasts were passaged twice prior to studies.

### Cell Treatment and miR-mimic Transfection

#### Cardiac Myocytes

Neonatal rat cardiac myocytes were cultured in DMEM containing 4.5 g/L D-glucose, 10% FBS, 100 U/mL Pen/Strep for the first 24 h. Dead cells were removed by rinsing in PBS, and culture medium was changed to 45% DMEM (1 g/L D-glucose), 45% Medium 199, 10% FBS, 100 U/mL Pen/Strep for 24 h. Subsequently, cells were cultured in medium containing 49% DMEM (1 g/LD-glucose), 49% Medium 199, 2% FBS, 100 U/mL Pen/Strep for 12 h. On day 3 following initial plating, culture medium was changed to serum-free medium containing 50% DMEM (1 g/L D-glucose), 50% Medium 199, 100 U/mL Pen/Strep for 12 h prior to initiation of cell treatment with Endothelin1 (ET1) (11, 18, 19). We selected ET1 because it had the most pronounced effect on gene expression (ANP, BNP, TGFB1-3, CTGF, and miR-29), when compared to Angiotensin II and Isoproterenol (data not shown).

Immunostaining was performed to confirm lack of fibroblast contamination of cardiac myocyte cultures (Supplemental Figure 1). For immunostaining, cells were fixed with 3.7% paraformaldehyde for 15 min at room temperature, washed with PBS and incubated with blocking solution for 1 h. Vimentin antibody (1:200; cat. no. D21H3; Cell Signaling Technology, Inc., Danvers, MA, USA) was added overnight at 4°C, followed by secondary antibody [Alexa-488 goat anti-rabbit IgG (H + L), 1:2,000; Cat.#A32731, Thermo Fisher Scientific] for 1 h. Hoechst (1:2,000; cat. no. H3569; Thermo Fisher Scientific, USA) was used to label nuclei. Slides were imaged using a Nikon confocal microscope with 20X objective.

We studied the following conditions: (1) Control, (2) ET1 treatment (ET1; 100 nM; Sigma) (20), (3) ET1 + N-acetyl-l-cysteine (NAC, 3.5 mM; Sigma). ET1 and NAC were added for 24 h; cell viability was confirmed prior to cell harvesting for gene and protein expression studies.

#### Cardiac Fibroblasts

Neonatal rat cardiac fibroblasts were expanded in DMEM containing 1 gm/L D-glucose, 10% FBS, 100 U/mL Pen/Strep. Fibroblasts were plated in six well-plates at a density of 0.5 × 10^6^ cells per well, and cultured in DMEM containing in 10% FBS for 24 h. Medium was subsequently changed to DMEM containing 2% FBS for 24 h. Medium was changed to serum-free DMEM for 8 h prior to treatment with ET1/TGFβ/miR-mimic (21, 22).

The miR-29a-mimic (Cat.#4464066) and control mimic (Control-mimic; Cat.#4464058) were purchased from Life Technologies, Inc. For miR-29a overexpression studies, cardiac fibroblasts were transfected with miR-29a-mimic (5 nM) or control-mimic (5 nM) for 6 h using Lipofectamine® RNAiMAX (Invitrogen; Thermo Fisher Scientific, Inc.) according to the manufacturer instructions. Culture medium was changed to serum-free DMEM prior to treatment with TGFβ1 or TGFβ2.

We studied the following conditions: (1) Control, (2) TGFβ1 (2 ng/mL; R&D Systems, Wiesbaden, Germany), (3) TGFβ2 (1 ng/mL; R&D Systems, Wiesbaden, Germany), (4) TGFβ1 + miR-29a-mimic, (5) TGFβ1 + control-mimic, (6) TGFβ2 + miR-29a-mimic; and (7) TGFβ2 + control-mimic, for 24 h. Cell viability was confirmed by microscopy prior to harvesting for gene and protein expression studies.

### HCM Mouse Models

The investigation conforms to the “Guide for the Care and Use of Laboratory Animals” published by the US National Institutes of Health (NIH 8th Edition, 2011), and was approved and monitored by the UCSF Laboratory Animal Resource Center. We studied two mouse models of sarcomeric HCM that have been well-characterized, namely the R403Q mutation in the α-myosin heavy chain gene (MyHC; *MYH6* gene) and the R92W mutation in the cardiac troponin T gene (TnT; *TNNT2* gene), that were kindly provided by Leinwand and Tardiff, respectively (3, 4). Mice were weaned and genotyped at the age of 4 weeks using PCR-amplified tail DNA. We studied male mice (mutants and littermate controls) at 5 and 24 weeks of age (redox studies, qPCR), and female mice at 5 weeks of age (qPCR of mouse LV tissue).

#### Adult Mouse Myocyte Isolation

Adult cardiac myocytes were dissociated as described previously (6). Briefly, mice were administered 100 IU heparin 10 min prior to euthanasia by cervical dislocation. Hearts were rapidly excised, cannulated via the aorta, and perfused in the Langendorf mode with a constant perfusion pressure of 80 mmHg. Hearts were perfused for 10 min using Ca^2+^-free Tyrode containing (in mM) NaCl (120), KCl (5.4), NaH_2_PO_4_ (1.2), NaHCO_3_ (20), MgCl_2_ (1.6), glucose (1 mg/ml), 2, 3-butanedione monoxime (BDM, 1 mg/ml), taurine (0.628 mg/ml), 0.9 mg/ml collagenase type 2 (Worthington, 299 U/mg), and gassed with 95% O_2_-5% CO_2_. The heart was then cut into small pieces and gently agitated, allowing myocytes to be dispersed in Ca^2+^-free Tyrode containing BSA (5 mg/L) for 10 min. Dispersed myocytes were filtered through a 150 μM mesh and gently centrifuged at 500 rpm for 30 s. Myocytes were then suspended in Tyrode containing gradually increasing amounts of Ca^2+^ (0.125–1 mM Ca^2+^) and stored in 1 mM Ca^2+^-containing Tyrode for microscopy studies.

### RNA Isolation and Polymerase Chain Reaction

Total RNA from mouse LV, left atrial (LA) appendage, rat cardiac myocyte cultures and rat cardiac fibroblast cultures was extracted using an RNA isolation Kit (Life Technologies) according to the manufacturer's instructions. RNA (1 μg of each sample) was reverse-transcribed into cDNA using cDNA Reverse Transcription kit (Applied Biosystems).

#### mRNA

Real-time RT-PCR for mRNA was performed using the TaqMan assay on a QuantStudio 7 Flex Real-Time PCR System (ThermoFisher, Inc.). Real-time RT-PCR was performed in duplicate, and samples were normalized to glyceraldehyde-3-phosphate dehydrogenase (GAPDH) expression. We tested the following genes involved in cardiac fibrosis. Profibrotic targets of miR-29, identified by TargetScan: collagen genes (*COL1A1, COL1A2*, and *COL3A1*) and elastin (*ELN*); connective tissue growth factor (*CTGF*); transforming growth factor-beta isoforms (*TGFB1-3*); transforming growth factor-beta receptors (*TGFBR1-2*). Additionally, we assessed genes involved in ROS scavenging [superoxide dismutase2 (*SOD2*), catalase (*CAT*)] and modulators of cardiac fibrosis [atrial natriuretic peptide (*ANP*), brain natriuretic peptide (*BNP*)].

#### miRNA

The real-time RT-PCR, TaqMan microRNA assay kit (Applied Biosystems) was used to quantify expression of mature miR-29a, miR-29b, and miR-29c in heart tissue, cardiac myocytes and cardiac fibroblasts. Real-time RT-PCR was performed in duplicate, and samples were normalized to U6 expression.

### Western Blot

Cardiac myocytes and fibroblasts were lysed in radio-immunoprecipitation assay lysis buffer (RIPA, Cat.#9806, Cell Signaling Technology) in the presence of protease inhibitors. Protein concentration was quantified using the bicinchoninic acid assay kit (Thermo Fisher Scientific). Denatured proteins (20 μg) from the samples were run on 4–12% NuPAGE™ Bis-Tris Gel (Thermo Fisher Scientific), following which proteins were transferred to polyvinylidene difluoride membranes. The membranes were subsequently blocked using 5% BSA for 1 h at room temperature, and incubated with TGFβ (Cat.#3711; 1:2,000; Cell Signaling) or collagen I (Cat.#ab233080; 1:3,000; Abcam) primary antibody, overnight at 4°C, washed several times, and then incubated with GAPDH (Cat.#5174; 1:20,000; Cell Signaling Technology) for 1 h at room temperature. Subsequently, a horseradish peroxidase-linked anti-rabbit IgG secondary antibody (Cat. #NA9340; 1:20,000; GE healthcare, Inc.) was incubated for 1 h at room temperature. An enhanced chemi-luminescence reagent (SuperSignal™ West Femto Maximum Sensitivity Substrate; Thermo Fisher Scientific) was used to visualize the protein bands. Semi-quantification of protein was conducted by comparison against the GAPDH bands using Image J software (version 1.48, National Institutes of Health, Bethesda).

### Quantification of TGFβ2 Secretion by ELISA

Release of TGFβ2 protein from cardiac myocytes into serum-free culture medium was determined using the Quantikine colorimetric sandwich enzyme-linked immunosorbent assay (ELISA) kit (R&D Systems), according to the manufacturer's instructions. ELISA was performed in duplicate. Absorbance at 450 nm was measured using a BioTek Synergy 2 multi-mode microplate reader. Protein concentration was calculated using a standard curve, and expressed as pg/10^6^ cells.

### Measurement of Reactive Oxygen Species and Cellular Redox Status

#### Rat Cardiac Myocyte Cultures

Neonatal rat cardiac myocytes were incubated with ET1 in the presence or absence of N-acetyl cysteine (NAC, 3.5 mM) for 24 h prior to imaging. Production of ROS in cultured cardiac myocytes was quantified using the cell-permeant fluorescence probes CM-H_2_DCFDA (DCF; Cat.#C6827, Thermo Fisher) and MitoSOX Red (Cat.#M36008), using an Olympus FV1000 MP microscope and 20X water immersion lens as described previously (23). Beating cardiac myocytes were incubated with DCF (5 μM) and MitoSOX Red (5 μM) for 30 min at 37°C, washed with PBS, prior to imaging at 37°C. We used an excitation wavelength of 800 nm. Emitted light was collected by three photomultiplier tubes fitted with bandpass filters. 512 × 512 pixel images were collected and image analysis was performed using Image J (NIH, http://rsb.info.nih.gov/ij/). Signal quantification was performed by drawing a region of interest (ROI) and calculating mean fluorescence intensity which was normalized to fluorescent area. DCF and Mitosox fluorescence was calculated from 10 randomly selected regions from three biological replicates (~300 cardiac myocytes).

#### Isolated Mouse Cardiac Myocytes

Cellular redox status was examined in freshly isolated, non-paced, adult mouse cardiac myocytes, as described previously (6). Experiments were performed at 37°C in a thermostatically controlled flow chamber mounted on the stage of an upright microscope (Nikon E600FN) attached to a multi-photon laser scanning system with excitation at 740 nm. Cells were suspended in Tyrode solution, pH 7.4, containing (in mM), NaCl (140), KCl (5), MgCl_2_ (1), HEPES (10), CaCl_2_ (1), and glucose (10). Monochlorobimane (MCB, 50 μM, blue λem 480 ± 20 nm) was loaded for 20 min on the stage of the microscope at 37°C to monitor reduced glutathione (GSH). Autofluorescence of NAD(P)H, namely total fluorescence collected at <490 nm was monitored separately. The acquired signal was calibrated by the addition of KCN (1 mM) for maximum reduction of existing NAD(P)H, followed by the addition of FCCP (5 μM) for maximum oxidation of NAD(P)H. Image analysis was performed using Image J software.

### Analysis of Gene Expression Data From HCM Patients

Gene expression data from publicly available microarray datasets, GSE36961 (mRNA) and GSE36946 (miRNA) was downloaded from the NCBI GEO database, and analyzed for differential gene expression. The microarray datasets were obtained from ventricular septal tissue of HCM patients undergoing myectomy at the Mayo Clinic (Rochester, MN), and from age/sex-matched donor hearts (control subjects) from the Sydney Heart Bank (24, 25). Information on HCM patient genotype/phenotype and control subjects was obtained from the publicly available Master's Thesis by Hebl (24).

Briefly, the raw data underwent quality control analysis. A microarray batch effect was noted in the data and adjusted for in the subsequent expression analysis. Data from 1 HCM patient that proved to be an outlier by principle component analysis, was excluded from further analysis. The fluorescence signal values were log_2_ converted and quantile normalized.

### Statistical Analysis

All results are expressed as mean ± S.D. unless otherwise noted. Two tailed Student's *t-*test was used to compare data from each mutant mouse to its respective littermate control, and to compare expression of select genes from human myectomy samples with control subjects. Experiments requiring comparison of ≥3 conditions were analyzed by ANOVA and Tukey's test. Graphs were generated using Graphpad Prism 7.04 software. For Ingenuity pathway analysis, mRNA data from 105 HCM patient samples was compared to 39 controls by ANOVA using the Partek Genomics Suite 7.0 platform. Differential expression was reported as fold change, and its statistical significance as *P*-value. Gene identifiers were updated to current HGNC/NCBI nomenclature. A *P* < 0.05 was considered statistically significant.

## Results

### Gene Expression in Rat Cardic Myocyte and Fibroblast Cultures

#### Cultured Rat Cardiac Fibroblasts Express Higher miR-29a Levels Than Cardiac Myocytes

**Figure 2:**
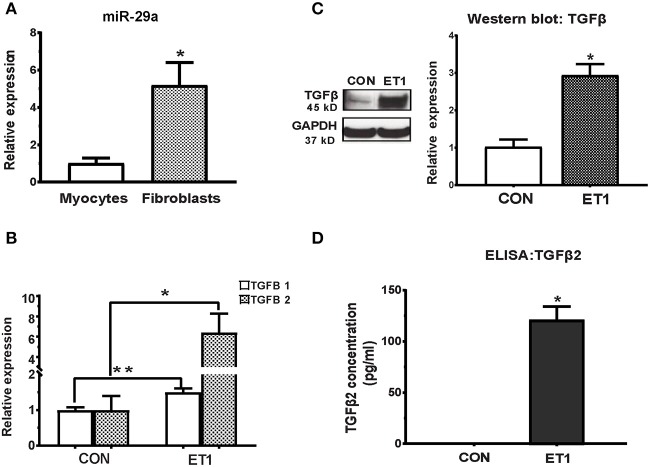
ET1 induced TGF-beta expression in neonatal rat cardiac myocyte cultures. **(A)** Real-time RT-PCR indicated 5-fold higher expression of miR-29a in cultured rat cardiac fibroblasts, when compared to rat cardiac myocytes. **(B)** ET1 increased *TGFB1/2* expression in cardiac myocytes. **(C)** ET1 increased TGFβ expression in cardiac myocytes. Image shows representative blot from one experiment and bar graph summarizes band intensity data from three experiments (data is expressed as mean ± S.D; *n* = 3; two tailed Student's *t-*test was used to determine significance; * *P* < 0.05). **(D)** TGFβ2 secretion was estimated by ELISA. ET1 (100 nM) stimulated TGFβ2 secretion by cardiac myocytes. [**(A,B,D)** CON, control; ET1, Endothelin1 (100 nM) treatment for 24 h. All data are expressed as mean ± S.D; *n* = 3 for each condition; each experiment was repeated three times. Two tailed Student's *t-*test was used to determine significance; **P < 0.01, *P < 0.05].

The miR-29 family, consisting of miR-29a, miR-29b, and miR-29c has been demonstrated to play an important role in cardiac (7), pulmonary (26), and renal (27) fibrosis. We found that expression of miR-29a was 5-fold higher in cultured rat cardiac fibroblasts when compared to cultured rat cardiac myocytes (Figure 2A). Furthermore, miR-29b and miR-29c expression was very low in rat cardiac myocytes when compared to miR-29a (data not shown), which led us to focus on miR-29a in our cell studies. Of note, low expression of miR-29b/c when compared to miR-29a, has been described previously in rat hearts (GEO: GSE62883, GSE56300).

#### ET1 Increases ROS and Stimulates TGFβ Secretion by Cardiac Myocytes

Fibrosis is a common pathologic and imaging feature in HCM (15, 28). In our recent study of MyHC and TnT mutant mouse hearts, before the onset of myocyte hypertrophy/fibrosis, pathway analysis of mRNA-seq data predicted up-regulation of ET1 signaling in both mutants (6). Since mutant sarcomeric proteins are expressed only in cardiac myocytes (not cardiac fibroblasts), and fibroblasts are the main effectors of cardiac fibrosis, we hypothesized involvement of a paracrine mechanism, namely TGFβ secretion by myocytes, in generation of ET1-mediated collagen deposition/fibrosis. We performed studies separately in neonatal rat cardiac myocyte and fibroblast cultures to test our hypothesis.

Treatment of cardiac myocytes with ET1 (100 nM) induced upregulation of *TGFB1-3, CTGF*, and suppression of miR-29a (Figure 2B and Table 1), but did not change expression of redox genes, *SOD2* and *CAT* (Table 1). ET1 also increased TGFβ protein expression (Figure 2C) and stimulated TGFβ secretion by cardiac myocytes (Figure 2D).

**Table 1:** Effects of ET1, ET1+NAC on gene expression in cardiac myocyte cultures.

**Genes**	**ET1**		**ET1 + NAC**	
	**FC**	***P*****-value vs. con**	**FC**	***P*****-value vs. ET1**
Natriuretic peptide A (*ANP*)	4.1	0.01	2.4	0.02
Natriuretic peptide B (*BNP*)	12.4	0.005	5.2	0.006
Transforming growth factor, beta 1 (*TGFB1*)	1.5	0.004	0.9	0.006
Transforming growth factor, beta 2 (*TGFB2*)	6.4	0.03	1.3	0.04
Transforming growth factor, beta 3 (*TGFB3*)	4.3	0.0001	2.4	0.001
Transforming growth factor, beta receptor 1 (*TGFBR1*)	1.6	0.004	0.8	0.01
Transforming growth factor, beta receptor 2 (*TGFBR2*)	0.7	0.1	0.6	0.8
Connective tissue growth factor (*CTGF*)	5.6	0.002	2.1	0.005
MicroRNA-29a (miR-29*a*)	0.6	0.01	1.7	0.006
Collagen type I alpha 1 chain (*COL1A1*)	1.4	0.005	1.1	0.009
Collagen type I alpha 2 chain (*COL1A2*)	1.7	0.0009	1.1	0.0008
Collagen type III alpha 1 chain (*COL3A1*)	1.3	0.03	1.0	0.03
Elastin (*ELN*)	1.4	0.03	0.6	0.004
Superoxide dismutase 2 (*SOD2*)	0.9	0.9	0.8	0.3
Catalase (*CAT*)	1.2	0.6	0.7	0.1

*Expression of miR-29a, select pro-fibrotic and redox genes in cardiac myocytes, following treatment with ET1 (100 nM) and ET1+NAC (3.5 mM)*.

*Data are expressed as linear fold change (FC) compared to control or ET1; n = 3 (biological replicates) for each condition; all experiments were repeated three times. One-way ANOVA and Tukey's test were used to compare ET1, ET1+NAC, and control*.

**Figure 3:**
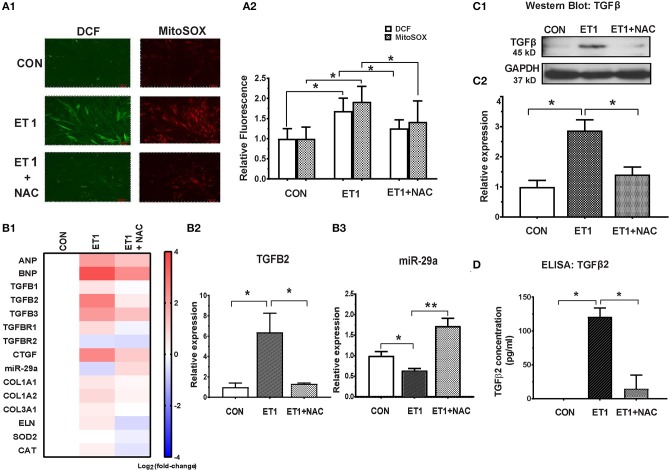
NAC abolished the ET1 stimulated increase in TGFβ expression and miR-29a suppression in neonatal rat cardiac myocyte cultures. **(A1)** Representative images of cardiac myocytes labeled with DCF and Mitosox, obtained by 2-photon microscopy. ET1 (100 nM) increased H_2_O_2_ (DCF fluorescence) and superoxide (MitoSOX fluorescence) in cardiac myocytes, which is prevented by NAC (3.5 mM). **(A2)** Summary of fluorescence data representing H_2_O_2_ (DCF) and superoxide (Mitosox) reveals increase in H_2_O_2_ and superoxide following ET1 (100 nM) treatment, which is abrogated by NAC (3.5 mM). DCF and Mitosox fluorescence was calculated from 10 randomly selected regions from three experiments (~300 cardiac myocytes). **(B1)** Heat map summarizing expression of miR-29a, select pro-fibrotic and redox genes in cardiac myocytes, following treatment with ET1 (100 nM) and ET1 + NAC (3.5 mM). ET1 suppressed miR-29a expression and increased expression of *TGFB1-3, CTGF*, and collagen genes. NAC increased miR-29a and blunted expression of pro-fibrotic genes following ET1 treatment. Differentially expressed genes are shown in graduated red (up-regulated) or blue (down-regulated) according to their log_2_ fold change compared to control (untreated cells). **(B2)** *TGFB2* was the most up-regulated TGFB gene in cardiac myocytes following ET1. *TGFB2* gene expression increased 6-fold following ET1 (100 nM) treatment. NAC antagonized the effect of ET1 and reduced *TGFB2* expression back to baseline levels in cardiac myocytes (linear fold change is presented). **(B3)** ET1 suppressed miR-29a expression, and NAC + ET1 increased miR-29a expression in cardiac myocytes (linear fold change is presented). **(C1)** Representative western blot image for TGFβ expression in cardiac myocytes (from one experiment) demonstrates that ET1 increases TGFβ, which is antagonized by NAC. **(C2)** Bar graph summarizing band intensity data from three experiments (biological replicates) following treatment with ET1 (100 nM) and ET1 + NAC (3.5 mM) shows that ET1 increases TGFβ protein expression in cardiac myocytes, which is blunted by NAC (data are expressed as mean ± S.D.; *n* = 3; one-way ANOVA and Tukey's test were used to compare ET1, ET1 + NAC, and control; *P < 0.05). **(D)** TGFβ2 secretion by cardiac myocytes was measured by ELISA. ET1 (100 nM) stimulated TGFβ2 secretion by cardiac myocytes, which was abrogated by NAC (3.5 mM). [**(A1,A2,B1–B3,D)** Data are expressed as mean ± S.D.; *n* = 3 for each condition; all experiments were repeated three times. One-way ANOVA and Tukey's test were used to compare ET1, ET1 + NAC, and control; **P < 0.01, *P < 0.05].

Two photon microscopy demonstrated increase in superoxide and hydrogen peroxide (H_2_O_2_) levels in cardiac myocytes following ET1 treatment (Figure 3A). Treatment with the ROS scavenger, N-acetyl-cysteine (NAC), a therapy predicted to be effective by IPA, only in 5 week-old TnT mutants (6), reduced cellular superoxide/H_2_O_2_, reduced expression of pro-fibrotic genes (*TGFB1-3, CTGF, COL1A1, COL1A2*, and *COL3A1*), and up-regulated miR-29a expression in cardiac myocytes (Figures 3B1–3 and Table 1). NAC also antagonized the ET1-induced increase in TGFβ expression (Figure 3C) and secretion by cardiac myocytes (Figure 3D).

#### TGFβ-Induced Collagen Expression in Cardiac Fibroblasts Is Prevented by a miR-29a-mimic

**Figure 4:**
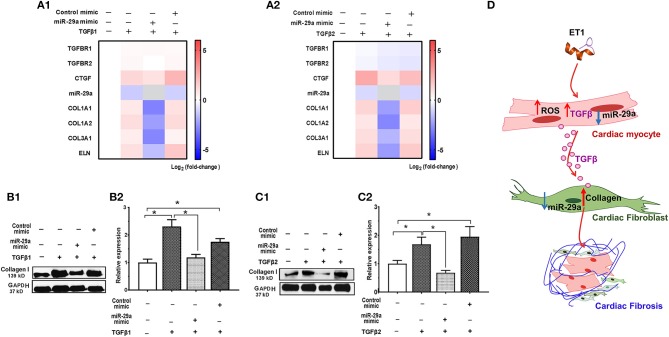
TGFβ-stimulated collagen expression in neonatal rat cardiac fibroblast cultures is prevented by miR-29a mimic. **(A1,A2)** Heat maps summarizing effect of TGFβ1 and TGFβ2 on expression of miR-29a and select pro-fibrotic target genes in cardiac fibroblasts. TGFβ1 and TGFβ2 suppress miR-29a expression and increase expression of pro-fibrotic genes, which is antagonized by the miR-29a-mimic, in cardiac fibroblasts. Differentially-expressed genes are shown in graduated red (up-regulated) or blue (down-regulated) according to their log_2_ fold change compared to control (untreated cells); miR-29a in the miR-29a-mimic condition is depicted in gray. **(B1,C1)** Representative western blot images for collagen type I following TGFβ1 and TGFβ2 treatment of cardiac fibroblasts (from one experiment) demonstrates that TGFβ1 and TGFβ12 stimulate collagen expression in cardiac fibroblasts, which is antagonized by a miR-29a-mimic. **(B2,C2)** Summary of western blot data for collagen type I following TGFβ1 or TGFβ2 treatment of cardiac fibroblasts from three experiments illustrates that TGFβ1 and TGFβ2 increase collagen type I expression in cardiac fibroblasts, which is inhibited by a miR-29a-mimic (data are expressed as mean ± S.D; *n* = 3. One-way ANOVA and Tukey's test were used to compare treatment conditions; *P < 0.05). **(D)** Schematic for stimulation of cardiac fibrosis following activation of ET1 signaling. ET1 promotes TGFβ secretion by cardiac myocytes, which downregulates miR-29a and de-represses its pro-fibrotic target genes in cardiac fibroblasts, leading to collagen deposition.

Since fibroblasts are the main effectors of cardiac fibrosis, we investigated the effect of ET1 and TGFβ on cardiac fibroblasts. ET1 did not change expression of *TGFB1-3*, miR-29a or its pro-fibrotic target genes in cardiac fibroblasts (Supplemental Figures 2A–C). In contrast, treatment with TGFβ1 (2 ng/ml) or TGFβ2 (1 ng/ml) downregulated miR-29a, upregulated *CTGF* and miR-29 targets (*COL1A1, COL1A2, COL3A1*, and *ELN*) in cardiac fibroblasts (Table 2, Figures 4A1,2, Supplemental Figures 3A,B). Notably, a miR-29a-mimic prevented upregulation of collagen genes (*COL1A1, COL1A2*, and *COL3A1*), elastin, and collagen expression in cardiac fibroblasts, following TGFβ1 and TGFβ2 treatment (Table 2, Figures 4A–C, Supplemental Figures 3A,B) highlighting the importance of miR-29a as a mediator of collagen deposition/fibrosis (27).

**Table 2:** Effects of TGFβ1, TGFβ2 and miR-29a mimic on gene expression in cardiac fibroblast cultures.

**Genes**	**TGFβ1**		**TGFβ1 + miR-29a mimic**		**TGFβ1 + control mimic**		**TGFβ2**		**TGFβ2 + miR-29a mimic**		**TGFβ2 + control mimic**	
	**FC**	**P vs. con**	**FC**	**P vs. TGFβ1**	**FC**	**P vs. con**	**FC**	**P vs. con**	**FC**	**P vs. TGFβ2**	**FC**	**P vs. con**
*TGFBR1*	1.1	0.12	1.1	0.7	1.1	0.1	0.8	0.4	0.7	0.2	0.7	0.1
*TGFBR2*	1.1	0.3	1.0	0.3	1.2	0.2	0.8	0.2	0.6	0.3	0.7	0.07
*CTGF*	2.3	0.002	1.8	0.09	4.8	0.02	6.5	3.2E-05	2.2	0.002	4.1	0.01
miR-29a	0.4	0.02	NA	NA	0.4	0.02	0.5	0.03	NA	NA	0.5	0.006
*COL1A1*	1.6	0.01	0.1	0.004	1.9	0.003	2.4	0.01	0.2	0.005	2.8	0.003
*COL1A2*	2.0	0.03	0.2	0.01	2.1	0.001	1.7	0.01	0.1	0.0007	1.6	0.02
*COL3A1*	1.5	0.02	0.1	0.004	1.3	0.01	2.0	0.01	0.3	0.0007	2.0	0.009
*ELN*	1.9	0.01	0.3	0.003	3.2	0.02	2.6	0.01	0.1	0.01	3.0	0.01

*Expression of miR-29a, select pro-fibrotic genes in cardiac fibroblasts, following treatment with TGFβ1 (2 ng/mL) or TGFβ2 (1 ng/mL) with/without miR-29a mimic. Data are expressed as linear fold change (FC); n = 3 for each condition; all experiments were repeated 3 times. One-way ANOVA and Tukey's test were used to compare TGFβ1/2, TGFβ1/2 + miR29a-mimic, TGFβ1/2 + control-mimic and control. TGFBR1, Transforming growth factor, beta receptor 1; TGFBR2, Transforming growth factor, beta receptor 2; CTGF, Connective tissue growth factor; miR-29a, microRNA 29a; COL1A1, Collagen type I alpha 1 chain; COL1A2, Collagen type I alpha 2 chain; ELN, Elastin*.

Taken together, our *in vitro* studies indicate that ET1 increases ROS and induces TGFβ secretion by cardiac myocytes, which downregulates miR-29a and de-represses its pro-fibrotic target genes in cardiac fibroblasts, leading to an increase in collagen expression in fibroblasts (Figure 4D).

### Redox Status in Mouse HCM

#### Allele-Specific Differences in Cardiac Myocyte Redox Status in HCM Mice

**Figure 5:**
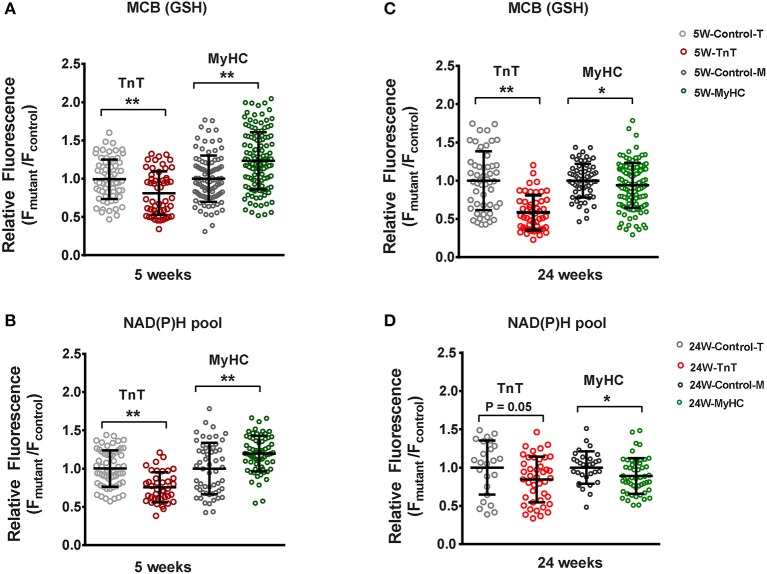
Two photon microscopy to assess redox status in mouse cardiac myocytes at 5 and 24 weeks of age. Cardiac myocytes were labeled with monochlorobimane (MCB; 50 μM, blue λ_em_ 480 ± 20 nm) at 37°C, to monitor reduced glutathione (GSH). Data from mutant myocytes is presented as fluorescence units normalized to littermate control myocyte data. NAD(P)H was assessed in non-labeled cells by measuring autofluorescence (total fluorescence collected at <490 nm). Potassium cyanide (KCN; 1 mM) and carbonyl cyanide-4-(trifluoromethoxy) phenylhydrazone (FCCP, 5 μM) were used to calibrate the NAD(P)H signal, permitting estimation of the NAD(P)H pool. All fluorescence recordings were obtained from non-paced cardiac myocytes. **(A)** TnT mutant myocytes had lower GSH (MCB signal), and MyHC mutant myocytes had higher GSH when compared to respective littermate control myocytes at 5 weeks of age. **(B)** NAD(P)H pool was lower in TnT mutant myocytes, and higher in MyHC mutant myocytes, when compared to respective littermate control myocytes at 5 weeks of age. **(C)** At 24 weeks of age, GSH (MCB signal) was lower in both mutant myocytes when compared to respective littermate control myocytes. **(D)** At 24 weeks of age, only MyHC mutant myocytes had statistically lower NAD(P)H pool levels, when compared to littermate control myocytes; a trend toward lower NAD(P)H pool levels were seen in TnT mutant myocytes when compared to littermate control myocytes (*p* = 0.05) (control-T, TnT-littermate control; TnT, TnT-mutant; Control-M, MyHC-littermate control; MyHC, MyHC-mutant) [Data are expressed as mean ± S.D. We present results from three mouse hearts (~20–40 myocytes per animal) in each mutant and control group. The two-tailed unpaired Student's *t-*test was used to compare TnT mutant myocytes and MyHC mutant myoytes with respective littermate control myocytes; *P < 0.05; **P < 0.01].

Since cellular redox status influenced TGFβ secretion by cardiac myocytes in our *in vitro* studies, we investigated cellular redox status in cardiac myocytes isolated from mutant and littermate control mouse hearts at 5 and 24 weeks of age. We performed two photon microscopy to assess reduced glutathione (GSH) and the NAD(P)H pool in isolated cardiac myocytes (Figure 5). Myocytes were labeled with monochlorbimane (MCB) to measure reduced glutathione (GSH), and cellular auto-fluorescence was used as an indicator of NAD(P)H.

TnT mutant myocytes had evidence of an oxidized redox environment, characterized by lower GSH, and NAD(P)H pool levels, when compared to littermate controls, at 5 weeks of age (Figures 5A,B). In contrast, MyHC mutant myocytes demonstrated a reduced redox environment, characterized by higher GSH and NAD(P)H pool at 5 weeks of age (6)—(Figures 5A,B).

At 24 weeks of age, both mutants demonstrated an oxidized redox environment. TnT mutants showed markedly lower GSH levels and a trend toward lower NAD(P)H pool levels (*p* = 0.05) when compared to littermate controls, whereas MyHC mutants demonstrated lower GSH and NAD(P)H pool levels, when compared to littermate controls (Figures 5C,D).

Taken together, our results indicate allele-specific, and disease stage-specific changes in cellular redox status in the two HCM mouse models.

### Gene Expression in Mouse and Human HCM

#### Allele-Specific Differences in Expression of miR-29a/b/c and Pro-fibrotic Genes in Left Ventricle of HCM Mice

We studied two mouse models of sarcomeric HCM with mutations that lead to cardiac phenotypes that span the spectrum of human HCM, namely malignant ventricular arrhythmias (R92W-TnT) (2, 3) and heart failure (R403Q-βMyHC) (4, 5, 16). The R403Q mutation in the *human* β-myosin motor domain, influences myosin-ATPase activity and leads to LV hypertrophy, severe heart failure (requiring heart transplantation), and/or arrhythmias (5, 29). In contrast, the R92W mutation in cTnT confers increased Ca^2+^ sensitivity to muscle fiber contraction, and predisposes to cardiac fibrosis and ventricular arrhythmias/sudden cardiac death in young individuals, in the absence of significant hypertrophy (30).

Our recent study comparing molecular phenotypes in 5 weeks old R92W-TnT and R403Q-MyHC *mice* with their respective littermate controls, demonstrated reduction in miR-29 expression only in mutant TnT whole hearts (6). However, it is unknown whether these differences are driven by gene expression in the LA and/or LV, and whether they persist later in disease course. In order to address these questions, we isolated the LV and LA appendage from each mouse heart, and assessed expression of *TGFB1-3*, miR-29a/b/c, and select target genes in these two chambers of mutant and littermate control mice at 5 and 24 weeks of age.

**Figure 6:**
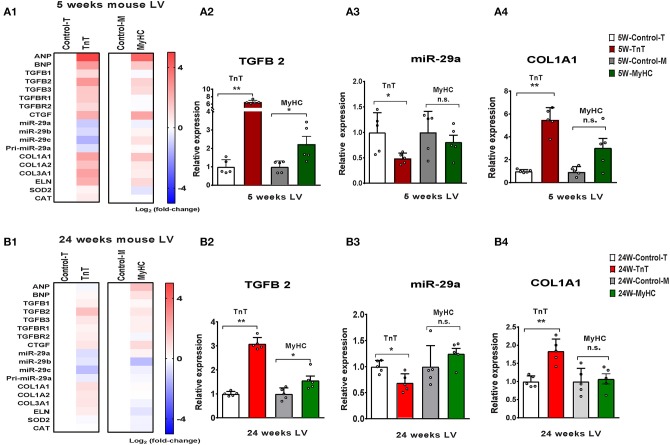
Allele-specific differences in expression of miR-29 family and pro-fibrotic genes in left ventricle of HCM mice at 5 and 24 weeks of age. **(A1**) Heat map summarizing expression data for miR-29a/b/c, select profibrotic and redox genes in the LV of TnT and MyHC mutant mice and respective littermate controls at 5 weeks of age. Expression of miR-29 family is significantly lower and profibrotic genes in significantly higher only in TnT mutant-LV when compared to littermate control-LV at 5 weeks of age. Differentially-expressed genes are shown in graduated red (upregulated) or blue (downregulated) based on their log_2_ fold-change compared to respective littermate controls. **(A2–4)** Summary for expression of *TGFB2*, miR-29a, and *Col1A1* in LV of TnT and MyHC mutants, and respective littermate controls at 5 weeks of age shows greater upregulation of *TGFB2* in TnT mutant-LV than MyHC mutant-LV, when compared to respective littermate control-LV. miR-29a suppression and increase in *COLl1A1* expression was only observed in TnT mutant-LV (linear fold change is presented). **(B1)** Heat map summarizing expression data for miR-29a/b/c, select pro-fibrotic and redox genes in LV of TnT and MyHC mutant mice and respective littermate controls at 24 weeks of age. Only TnT mutant-LV demonstrate downregulation of miR-29 family at 24 weeks of age. Differentially expressed genes are shown in graduated red (upregulated) or blue (downregulated) based on their log_2_ fold change compared to respective littermate controls. **(B2–4)** Summary for expression of *TGFB2*, miR-29a, and *COL1A1* in LV of TnT and MyHC mutant mice, and respective littermate controls at 24 weeks of age shows greater upregulation of *TGFB2* in TnT mutant-LV than MyHC mutant-LV, when compared to respective littermate controls. Expression of miR-29a/b/c is lower and *COL1A1* gene expression is higher in TnT mutant-LV, but not MyHC mutant-LV when compared to respective littermate controls (linear fold change is presented) (control-T, TnT-littermate control; TnT, TnT-mutant; Control-M, MyHC-littermate control; MyHC, MyHC-mutant). Data are expressed as mean ± S.D.; *n* = 5 in each group. The two-tailed unpaired Student's *t-*test was used to compare TnT mutants and MyHC mutants with respective littermate controls; **P < 0.01; *P < 0.05; n.s., non-significant).

We observed upregulation of *TGFB2* and *CTGF* expression in the LV of both mutant mice at 5 and 24 weeks of age, but *TGFB1* gene expression was increased in TnT mutant-LV, but not MyHC mutant-LV, at both time points (Figures 6A,B and Table 3). Furthermore, miR-29a/b/c was downregulated, and its pro-fibrotic target genes (*COL1A1, Col1A2, COL3A1*, and *ELN*) were upregulated in TnT mutant-LV, but not MyHC mutant-LV at both time points (Figures 6A,B and Supplemental Figure 4).

**Table 3:** Gene expression in mutant mouse-LV compared to littermate control-LV at 5 and 24 weeks of age.

**Genes**	**5 week male mice LV**				**24 week male mice LV**			
	**TnT**		**MyHC**		**TnT**		**MyHC**	
	**FC**	***P*****-value vs. con**	**FC**	***P*****-value vs. con**	**FC**	***P*****-value vs. con**	**FC**	***P*****-value vs. con**
*ANP*	29	0.04	16.3	0.01	0.9	0.3	3.1	7.4E-05
*BNP*	6.2	1.1E-05	2.7	0.1	1.2	0.1	1.7	0.004
*TGFB1*	2.0	0.001	0.9	0.7	1.3	0.004	1.2	0.4
*TGFB2*	6.5	6.4E-08	2.2	0.04	3.1	9.6E-06	1.6	0.03
*TGFB3*	2.7	1.0E-05	1.9	0.1	1.6	0.002	1.3	0.01
*TGFBR1*	2.6	8.4E-05	0.9	0.8	1.3	0.04	1.3	0.07
*TGFBR2*	2.0	2.2E-06	1.0	0.9	1.4	0.06	0.9	0.2
*CTGF*	4.4	0.0004	5.2	0.001	1.6	0.0003	2.0	0.002
miR-29a	0.5	0.03	0.8	0.4	0.6	0.01	1.2	0.2
miR-29b	0.6	0.03	1.2	0.5	0.7	0.03	0.4	0.06
miR-29c	0.3	0.04	1.7	0.04	0.4	0.004	0.8	0.3
Pri-miR-29a	0.6	3.5E-05	1.1	0.7	0.8	0.03	0.9	0.4
*COL1A1*	5.5	0.0006	3.0	0.06	1.8	0.002	1.1	0.7
*COL1A2*	3.1	1E-05	1.5	0.2	1.6	0.004	1.0	0.9
*COL3A1*	5.5	0.0002	1.4	0.2	1.6	0.0002	0.8	0.5
*ELN*	4.2	4.1E-07	2.0	0.2	1.4	0.02	0.7	0.06
*SOD2*	1.3	0.06	0.7	0.06	0.9	0.4	0.8	0.1
*CAT*	1.4	0.2	1.0	0.7	1.0	0.8	0.8	0.9

*Expression data for miR-29a/b/c, select profibrotic and redox genes in the LV of TnT and MyHC mutant mice and respective littermate controls at 5 and 24 weeks of age. Data are expressed as mean ± S.D.; n = 5 in each group. The 2-tailed unpaired Student's t test was used to compare TnT mutants and MyHC mutants with respective littermate controls. ANP, Natriuretic peptide A; BNP, Natriuretic peptide B; TGFB1, Transforming growth factor, beta 1; TGFB2, Transforming growth factor, beta 2; TGFB3, Transforming growth factor, beta; TGFBR1, Transforming growth factor, beta receptor 1; TGFBR2, Transforming growth factor, beta receptor 2; CTGF, Connective tissue growthfactor; miR-29a, microRNA-29a; COL1A1, Collagen type I alpha 1 chain; COL1A2, Collagen type I alpha 2 chain; ELN, Elastin*.

#### Gene Expression in Left Atrial Appendage of HCM Mice

We did not find a statistically significant difference in the expression of *TGFB1-3*, miR-29a/b/c or its target genes in LA appendages from TnT and MyHC mutants compared to respective littermate control mice at 5 and 24 weeks of age (Supplemental Figure 5).

#### Human Data: Gene Expression in Septal Myectomy Tissue Compared to Donor Heart Tissue (Controls)

**Figure 7:**
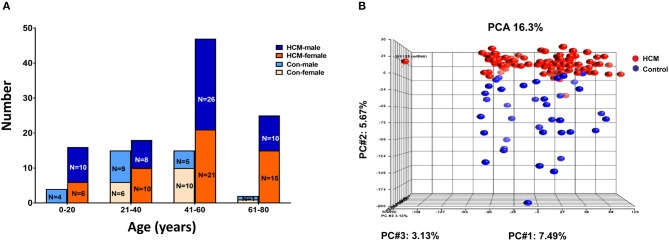
Microarray data from HCM patients undergoing myectomy compared to control (healthy organ donor) hearts. **(A)** Age and sex distribution of HCM patients and controls. **(B)** PCA plot represents the results of a principal component analysis of all samples' normalized log_2_ fluorescence values, wherein each dot represents a single sample, its color indicates HCM (red) or control (blue) status. Each of the plot's three dimensions separates the samples based on a subset of all the microarray's genes, selected by correlation. The X-axis gene set represents the highest percentage of overall variation, followed by Y and Z in that order. One HCM sample on the X-axis (far left) is an outlier and was excluded from normalization of the ANOVA signal values. The Y-axis depicts a good, albeit not perfect, separation of the two biological classes, which ANOVA defines as the genes that distinguish HCM hearts from control hearts.

We analyzed publicly available microarray data for mRNAs (GSE36961) and miRNAs (GSE36946) from ventricular septal tissue, obtained at the time of surgical myectomy from HCM patients, and control subjects (24, 31). Donor heart tissue (LV free wall or LV side of the interventricular septum) from the Sydney Heart Bank (24) was used as controls. Age of HCM patients ranged from 9 to 78 years. Patients had a severe cardiac HCM phenotype, with maximum LV wall thickness of 2.2 ± 0.7 cm, and LV gradients of 71.5 ± 47.4 mmHg (24). Genotyping was performed in 100/107 HCM patients undergoing myectomy, revealing pathogenic variants in MYH7 (*n* = 17), MYBPC3 (*n* = 24), TNNT2 (*n* = 4), TNNC1 (*n* = 1), TPM1 (*n* = 2), MYL2 (*n* = 3), and MYH6 (*n* = 1) (24); no causal mutation was identified in the remainder (*n* = 48). Figure 7A illustrates the age and sex distribution of HCM patients and control subjects. We excluded 1 HCM patient based on the results of our principle component analysis (PCA), leading to inclusion of 105 patients/39 controls from the mRNA dataset, and 106 patients/20 controls from the miRNA datasets (Figure 7B) in our analysis.

#### Expression of Genes Involved in Cardiac Fibrosis

**Figure 8:**
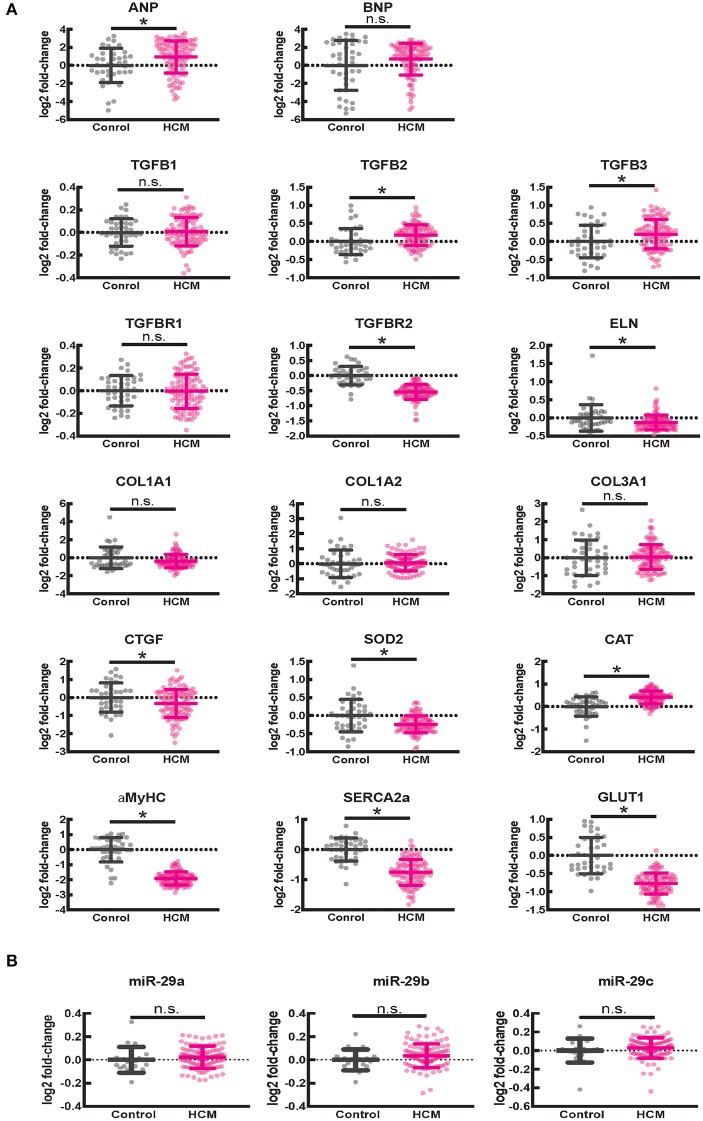
Gene expression in human ventricular septal tissue of HCM patients undergoing myectomy compared with control (healthy organ donor) hearts. **(A)** Summary of mRNA data (select pro-fibrotic and redox genes) from 105 HCM patients compared to data from 39 controls shows upregulation of *TGFB2* and *TGFB3* and downregulation of *TGFBR2, ELN*, α*MYHC, SERCA2a*, and *GLUT1* genes. **(B)** Summary of data for miR-29a/b/c from 106 HCM patients compared with data from 20 controls shows no difference in expression of miR-29a/b/c between HCM patients and controls. *P < 0.05.

Since fibrosis is a prominent feature in the hypertrophied interventricular septum, we examined several genes involved in cardiac fibrosis. *TGFB2* and *TGFB3* were significantly upregulated, and *TGFBR2* was downregulated in HCM, but there was no difference in expression of *CTGF, TGFB1*, miR-29/a/b/c and its pro-fibrotic targets (collagen genes, elastin), in myectomy samples compared to donor heart tissue (Figures 8A,B). Next, we examined expression of renin-angiotensin-aldosterone system (RAAS) genes. The angiotensin converting enzyme 2 (*ACE2*) gene, a negative regulator of RAAS (32) was the most upregulated transcript in the HCM patient dataset (Table 4). Other members of the RAAS system, namely renin-binding protein (*RENBP*), renin receptor mannose-6-phosphate/insulin-like growth factor 2 receptor (*IGF2R*), angiotensinogen (*AGT*) and angiotensin II receptor type 1 (*AGTR1*) were significantly downregulated (Table 4), suggesting activation of compensatory anti-hypertrophic/anti-fibrotic pathways in human obstructive HCM.

**Table 4:** Expression of RAAS genes in human myectomy tissue from HCM patients compared with control (healthy donor) hearts.

**Gene**	**FC**	**Adjusted *P*-value**
Angiotensin converting enzyme 2 (*ACE2*)	3.5	2.2E-24
Renin-binding protein (*RENBP*)	1.1	5.7E-05
Insulin-like growth factor 2 receptor (*IGF2R*)	0.8	1.1E-03
Angiotensinogen (*AGT*)	0.8	1.2E-04
Angiotensin II receptor type 1 (*AGTR1*)	0.8	2.0E-13
Angiotensin converting enzyme (*ACE*)	1.0	0.09
Angiotensin II receptor type 2 (*AGTR2*)	1.0	0.9
Angiotensin II receptor associated protein (*AGTRAP*)	0.9	0.4
Renin (*REN*)	0.9	0.9
Aldosterone synthase (*CYP11B2*)	1.0	0.4
(Pro)renin receptor (*ATP6AP2*)	0.9	0.7
Cathepsin D (*CTSD*)	0.9	0.5
Chymase A (*CMA1*)	0.9	0.5

We observed lower expression of *SOD2* and higher expression of catalase in myectomy tissue when compared to donor heart tissue. Furthermore, α*MYHC, SERCA2a*, and *GLUT1* were downregulated, and *ANP* was upregulated, compared to donor heart tissue (Figure 8A), confirming activation of the fetal gene program (33) in human obstructive HCM.

#### Signaling Pathways

**Figure 9:**
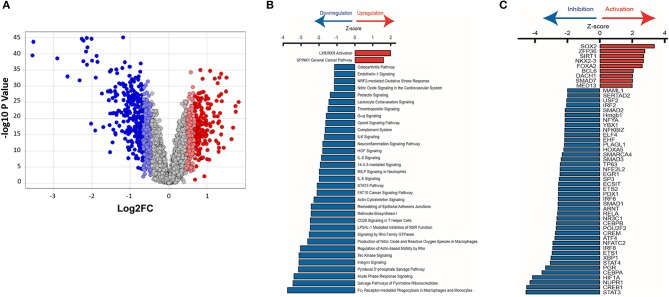
Ingenuity pathway analysis of mRNA microarray data obtained from HCM patient myectomy tissue compared to control (healthy donor) heart tissue. **(A)** This volcano plot represents the ANOVA results of HCM vs. controls, depicting genes' log_2_ fold changes on the X-axis and their –log_10_ *P*-values on the Y-axis. Each dot in the figure represents a gene and is colored according to the standard deviation (SD) of that gene's log fold change from the mean. Genes with greater than 2SD up or down were used in the IPA analysis. **(B)** IPA identified pathways that are significantly (*P* < 0.032) up-regulated (*Z*-score > 0) or down-regulated (*Z*-score < 0) in HCM patients compared to control subjects. **(C)** Identification of upstream transcriptional regulators that are upregulated and downregulated in HCM patients compared to control subjects.

We used Ingenuity Pathway Analysis (IPA, Qiagen) to predict dysregulated signaling pathways and upstream transcriptional regulators using the mRNA dataset. The red and blue dots in the volcano plot reflect the gene values used for IPA (Figure 9A). We used a log_2_ fold change cutoff of 2SD (standard deviations) up or down, which corresponds to a linear fold change of ~1.37, for IPA.

Pathway analysis predicted activation of the liver X receptor/retinoid X receptor (LXR/RXR) pathway (*Z* = 2.0; *P* = 0.00006; Figure 9B), and downregulation of pro-hypertrophic ET1 signaling (*Z* = −1.1; *P* = 0.007), TGFβ signaling (*Z* = −2.2; *P* = 0.4), and cardiac hypertrophy signaling (*Z* = −1.5; *P* = 0.5) in human HCM hearts compared to control hearts (data not shown).

#### Upstream Transcriptional Regulators

We used IPA to predict transcription factors that were up-regulated and down-regulated in human myectomy tissue, when compared to donor heart tissue. Transcription factors involved in development (SOX2, NKX2-3, and DACH1), inflammation (ZFP36), metabolism (SIRT1, FOXA2), and fibrosis-suppression (SMAD7) were predicted to be upregulated (*Z* > 2) in HCM patients, when compared to controls (Figure 9C). Transcription factors involved in development/immune response (STAT3), metabolism/mitochondrial function (CREB1), cellular stress response/fibrosis (NUPR1), and cellular response to hypoxia (HIF1) were predicted to be most downregulated (*Z* > −4) in human myectomy samples, when compared to donor heart tissue (Figure 9C).

## Discussion

In this study, we demonstrate marked differences in expression of pro-fibrotic genes/pathways in mouse and human HCM. Gene expression analysis revealed activation of anti-hypertrophic and antifibrotic genes/pathways selectively in human myectomy tissue, which is novel. Our results in cardiac myocytes and fibroblasts provide insights into mechanisms whereby hypertrophy induction in cardiac myocytes promotes collagen expression in fibroblasts. We confirmed allele-specific differences in expression of *TGFB*, miR-29 family, and its profibrotic target genes at both early and established disease stage in two mouse models of sarcomeric HCM. Our studies of redox and gene expression suggest that allele-specific differences in redox and signaling pathways could contribute to allele-specific differences in upregulation of profibrotic genes in mouse HCM.

### Human HCM

Hypertrophic cardiomyopathy is classified as non-obstructive, labile obstructive or obstructive, based on pressure gradients obtained by Doppler-echocardiography, at the level of the LV outflow tract and mid-LV cavity. Non-obstructive HCM is characterized by peak gradient <30 mm Hg at rest and stress, labile-obstructive HCM by peak gradient <30 mm Hg at rest, ≥30 mm Hg with stress, and obstructive HCM with peak gradient ≥30 mm Hg at rest and stress (34). Symptomatic HCM patients with obstructive HCM are referred for myectomy to relieve LV obstruction.

The ventricular septum is usually the most hypertrophied region and is a frequent location for late gadolinium enhancement, reflecting replacement fibrosis, by cardiac magnetic resonance imaging (35). Septal myectomy samples often demonstrate both replacement and interstitial fibrosis (36) by histopathology. To our surprise, analysis of gene expression in the excised hypertrophied ventricular septum revealed no difference in expression of *CTGF, TGFB1*, miR-29a/b/c, and its pro-fibrotic target genes (collagen genes, elastin) between HCM patients and control subjects. Notably, IPA predicted downregulation of pro-fibrotic/pro-hypertrophic TGFβ/ET1 signaling and several RAAS genes, along with marked upregulation of the LXR/RXR pathway, which is anti-fibrotic, and anti-hypertrophic. These results suggest that other mechanisms such as ischemia (30), cell death, and inflammation could underlie the fibrosis observed in the hypertrophied ventricular septum in human HCM. Interestingly, we did not find differences in expression of RAAS genes, and IPA did not predict upregulation of the LXR/RXR pathway in HCM *mouse* models that lack LV outflow tract obstruction at early and late disease stage (data not shown). These results suggest that LXR/RXR pathway activation may be a compensatory response (37) to sustained LV obstruction, which is characteristic of obstructive HCM.

The liver X receptor (LXR) and retinoic acid receptor (RXR) are nuclear receptors that form obligate heterodimers and bind to LXR response elements (LXREs) in the nucleus (38). In the heart, LXR expression has been reported to be 10- to 15-fold higher in non-myocytes (fibroblasts, endothelial cells) when compared to cardiac myocytes (39). RXR is involved in cardiac development and LXR has been implicated in the adaptive metabolic response to hypertrophic stress (40). Studies in mouse models reveal that LXR regulate cell survival through ROS inhibition, antagonize TGFβ-mediated collagen synthesis, promote angiogenesis, and suppress pro-inflammatory NFκB signaling, which are all cardio-protective (39, 41, 42). But very little is known about the role of LXR/RXR pathway in the genesis of the cardiac HCM phenotype.

### Differential Expression of TGF-Beta Isoforms

Fibrosis is frequent in HCM patients, and contributes to arrhythmias and heart failure (27, 43, 44). *TGFB1* was up-regulated in TnT mutant-LV and ET1-treated cardiac myocytes, but not in human myectomy tissue or MyHC mutant-LV. The TGF-beta isoform upregulated in both mouse and human HCM, as well as ET1-treated cardiac myocytes, was *TGFB2*. Differential expression of TGFB isoforms has also been reported in DCM mice (45), but the mechanisms underlying differential regulation of *TGFB1* and *TGFB2* genes, and the implications of these differences on HCM pathophysiology is unknown. Our *in vitro* studies of gene and collagen expression reveal that both TGFβ1 and TGFβ2 suppress miR-29a, de-repress its profibrotic target genes, and stimulate collagen expression in cardiac fibroblasts.

### Association Between miR-29 and Cardiac Remodeling

A previous study of 41 HCM patients revealed significant increases in plasma levels of miR-29a and a positive association with cardiac hypertrophy and fibrosis, assessed by cardiac magnetic resonance imaging (8). This result was surprising, because tissue fibrosis has been associated with suppression of miR-29 expression, which should have resulted in a negative association between miR-29 and cardiac fibrosis. These clinical results could be explained by a recent study that performed over-expression and knockdown of miR-29 family in cardiac myocytes (46). Sassi et al. found that increasing miR-29 family in cardiac myocytes led to cardiac hypertrophy, and inhibition or genetic deficiency of miR-29 family protected against cardiac hypertrophy and fibrosis in a mouse model of chronic cardiac pressure overload. The authors proposed that de-repression of Wnt signaling underlies the pro-hypertrophic and pro-fibrotic effects of increased levels of miR-29 family in cardiac myocytes (46).

In our study, miR-29 family expression was lower in TnT mutant-LV at 5 and 24 weeks of age, but no difference was observed in MyHC mutant-LV at these two time points. Interestingly, TnT mutant-LV mass has been reported to be lower than controls at both time points (47), which may contribute to lower cardiac miR-29 family levels. We found no difference in expression of miR-29a/b/c and its pro-fibrotic target genes in ventricular septal tissue obtained from patients with obstructive HCM undergoing myectomy for relief of LV outflow tract/mid-cavity obstruction. The discrepancy between our result and the clinical plasma-miRNA study which reported a positive association between miR-29a and cardiac hypertrophy and fibrosis, may be due to differences in cardiac physiology. The mean value for the LV outflow tract gradient, which is used to determine presence of LV outflow tract obstruction, was 18 ± 21 mmHg, suggesting that most of the patients in the clinical plasma-miRNA study had non-obstructive HCM (8). In contrast, the HCM patients in our study had obstructive HCM which results in chronic increase in left ventricular afterload. Interestingly, Sassi et al. reported dynamic regulation of miR-29 family expression following transverse aortic constriction (TAC), which increases left ventricular afterload (46). They found a prominent increase in cardiac myocyte expression of miR-29 in the first 48 h after TAC surgery, and downregulation thereafter, which could help explain our results in myectomy tissue. It is also possible that circulating miRNAs may not correlate with miRNA expression in tissues like the heart. Investigation of plasma miR-29 family levels in conjunction with gene expression studies in patients undergoing myectomy would be helpful to define the relationship between cardiac expression and plasma levels of the miR-29 family.

### Allele-Specific and Disease Stage-Specific Differences in miR-29a/b/c and Pro-fibrotic Gene Expression

We observed 5-fold higher levels of miR-29a in cultured rat cardiac fibroblasts, when compared to rat cardiac myocytes, which is similar to the results from a previous study (7). In contrast, Sassi et al. found that miR-29 family levels were 6-fold lower in freshly isolated cardiac fibroblasts, when compared to cardiac myocytes (46), and increased with cell culture. A likely explanation for this difference is that long term culture of cardiac fibroblasts on stiff substrates like tissue culture plastic or glass coverslips activates them to myofibroblasts (48).

In our *in vitro* study that employed cultured rat cardiac fibroblasts and myocytes, the pro-hypertrophic cytokine ET1 increased superoxide and H_2_O_2_ levels, suppressed miR-29a/b/c expression, and induced TGFβ secretion by cardiac myocytes. Interestingly, ET1 was predicted (by IPA) to be upregulated in *both* mutants at 5 weeks of age (6), but TGFβ signaling was predicted to be upregulated only in TnT mutants. Furthermore, miR-29a/b/c was suppressed only in TnT mutants who also demonstrated an oxidized redox environment at 5 weeks of age. In contrast, MyHC mutants exhibited a reduced redox environment and demonstrated similar miR-29 expression as littermate controls at 5 weeks of age. Based on our *in vitro* results of ET1 increasing cellular ROS levels and *TGFB1-3* expression, we speculate that TnT mutants that demonstrate an oxidized redox environment are more susceptible to ET1-stimulated activation of TGFβ signaling, which suppresses miR-29 and leads to increase in collagen gene expression/cardiac fibrosis. Our hypothesis is supported by identification of the antioxidant NAC, as a potential therapy by IPA, in TnT mutants, but not MyHC mutants at 5 weeks of age (6).

In addition to TGFβ signaling, hedgehog, NFκB signaling, C-Myc, CCAAT/enhancer-binding protein-α (CEBPA), and ROS have been demonstrated to regulate miR-29 expression (49–51). Our study did not detect a difference in miR-29a/b/c expression between human myectomy tissue and donor heart tissue, which was used as controls. Based on results of a prior study of HCM patients which suggested that obstructive HCM is associated with greater oxidative stress than non-obstructive HCM (52), we expected higher myocardial miR-29 levels in human myectomy tissue (51). The mechanism underlying our result could be downregulation (*Z* > −3) of CEBPA, an inducer of miR-29 expression, in human myectomy samples. We speculate that the net effect of oxidative stress, CEBPA downregulation and lack of activation of TGFβ signaling, could have resulted in no difference in miR-29 family/target gene expression between HCM patients and controls, observed in our study.

## Limitations

We focused on the miR-29/TGFβ signaling pathway, and did not investigate the LXR-RXR pathway in HCM mouse models or *in vitro*. We limited our proof-of-principle studies to two mouse models of HCM, in order to examine allele-specific and stage-specific differences in redox and gene expression. Studies in additional experimental models (53) would be helpful to ascertain whether HCM pathophysiology is gene-specific or mutation-specific. We used publicly available microarray data, and did not have access to human myectomy tissue to perform qPCR or Western blot for confirmation of IPA predictions. Since human myectomy tissue was obtained from the septum, gene expression in this region may not be representative of the whole heart. Furthermore, the contribution of cardiac myocytes, fibroblasts, and endothelial cells to differences in gene expression observed in human heart tissue could not be assessed because RNA was obtained from homogenized heart tissue, without cell dissociation. Lastly, we obtained genotype information for the myectomy population from the publicly available Master's Thesis by Hebl (24), but did not have access to genotype data from individual patients. Hence we were unable to examine associations between HCM patient genotype and gene expression, in this study.

## Conclusion

In summary, we found marked differences in expression of *TGFB1*, miR-29 family and its pro-fibrotic target genes in two mouse models of sarcomeric HCM, and between mouse and human HCM. Only human myectomy tissue demonstrated upregulation of antihypertrophic/antifibrotic genes/pathways. Allele-specific differences in myocyte redox status and signaling pathways could contribute to the observed differences in expression of miR-29 family/profibrotic genes in the 2 mouse models of HCM, and differences in cardiac physiology could underlie predicted activation of compensatory anti-fibrotic/anti-hypertrophic signaling/genes selectively in human HCM. Parallel studies in mouse and human HCM are needed to define HCM pathophysiology and identify therapies that prevent/regress the cardiac HCM phenotype in humans.

## Data Availability Statement

The datasets generated for this study can be found in the GSE36961 (mRNA) and GSE36946 (miRNA).

## Ethics Statement

Ethical review and approval was not required for the study on human participants in accordance with the local legislation and institutional requirements. Written informed consent for participation was not required for this study in accordance with the national legislation and the institutional requirements. The animal study was reviewed and approved by the investigation conforms to the Guide for the Care and Use of Laboratory Animals published by the US National Institutes of Health (NIH 8th Edition, 2011) and was approved and monitored by UCSF Laboratory Animal Resource Center.

## Author Contributions

YL, MA, and RF were responsible for overall study design and data analysis. YL designed, performed, and analyzed rat and mouse studies. RF and CT analyzed microarray data. JA performed two-photon studies in rat myocytes, and contributed to rat myocyte/fibroblast study design. SV performed two photon microscopy studies in isolated mouse cardiac myocytes. YG performed mouse myocyte isolation. YG and GG maintained the mouse colonies and performed mouse genotyping. RK assisted with rat and mouse studies. JT and LL provided transgenic mouse breeders for colony establishment. YL, RF, CT, MA, SD, VH, LL, JT, and JO were involved in manuscript preparation. All authors reviewed and approved the manuscript.

### Conflict of Interest

LL is a stockholder and founder of MyoKardia, Inc. The remaining authors declare that the research was conducted in the absence of any commercial or financial relationships that could be construed as a potential conflict of interest.

## Supplementary Material

Supplemental Figure 1 Vimentin immunofluorescence microscopy. Immunostaining for vimentin demonstrates no fibroblast contamination of neonatal rat cardiac myocyte cultures. **(A1–A3)** Representative microscopy images of a neonatal rat cardiac fibroblast culture shows co-localization of Hoechst and vimentin-labeling. **(B1–B3)** Representative microscopy images of a neonatal rat cardiac myocyte culture shows nuclear labeling with Hoechst (blue), but no vimentin-positive cells.

Supplemental Figure 2 ET1 has no effect on miR-29a and pro-fibrotic gene expression in cultured neonatal rat cardiac fibroblasts. **(A–C)** Summary of gene expression of *TGFB2, CTGF*, collagen genes, and miR-29a show no difference in expression between untreated (control) and fibroblasts treated with ET1 (100 nM for 24 h) [CON, control; ET1, endothelin1 (100 nM) treatment for 24 h. Data are expressed as mean ± S.D; *n* = 3 (biological replicates); all experiments were repeated three times. Two tailed Student's *t-*test was used to determine significance; n.s., non-significant].

Supplemental Figure 3 TGβ1/2 stimulated collagen expression in neonatal rat cardiac fibroblast cultures is antagonized by miR-29-mimic. Cardiac fibroblasts were either treated with 5 nM concentration of either miR-29a-mimic or control-mimic for 6 h, prior to stimulation with TGFβ1 or TGFβ2 for 24 h. **(A)** miR-29a-mimic suppressed TGFβ1-stimulated expression of profibrotic miR-29 targets, but not *CTGF*, which is not a miR-29 target gene (linear fold change is presented). **(B)** miR-29a-mimic suppressed TGFβ2-stimulated expression of profibrotic miR-29 targets, but not *CTGF* (linear fold change is presented) [data are expressed as mean ± S.D.; *n* = 3 (biological replicates) for each condition; all experiments were repeated three times. One-way ANOVA and Tukey's test were used to compare treatment conditions; ^*^*P* < 0.05].

Supplemental Figure 4 Allele-specific differences in expression of miR-29a, *ANP, TGFB1/2, COL1A1* in LV of female HCM mice/littermate controls at 5 weeks of age. **(A–E)** Allele-specific differences in expression of *ANP, TGFB1/2*, miR-29a, and *COL1A1* in female TnT and MyHC mutant mouse -LV and littermate control-LV at 5 weeks of age (Control-T, TnT-littermate control; TnT, TnT-mutant; Control-M, MyHC-littermate control; MyHC, MyHC-mutant) **(**data are expressed as mean ± S.D.; *n* = 6 in each group. The two-tailed unpaired Student's *t-*test was used to compare TnT mutants and MyHC mutants with respective littermate controls; ^*^*P* < 0.05; n.s., non-significant).

Supplemental Figure 5 Gene expression in left atrial appendage of HCM mice and littermate controls at 5 and 24 weeks of age. **(A,B)** No difference in expression of miR-29a/b/c, select pro-fibrotic and redox genes between left atrial appendage of mutant mice and littermate controls at 5 and 24 weeks of age (control-T, TnT-littermate control; TnT, TnT-mutant; Control-M, MyHC-littermate control; MyHC, MyHC-mutant) (data are expressed as mean ± S.D.; *n* = 5 for each group. The two-tailed unpaired Student's *t-*test was used to compare TnT mutants and MyHC mutants with respective littermate controls; n.s., non-significant).
