# Supplementary figures and images for: Differences in microRNA-29 and Pro-fibrotic Gene Expression in Mouse and Human Hypertrophic Cardiomyopathy
Source: Front Cardiovasc Med. 2019 Dec 17;6:170. doi: 10.3389/fcvm.2019.00170 (PMC6928121; doi:10.3389/fcvm.2019.00170)

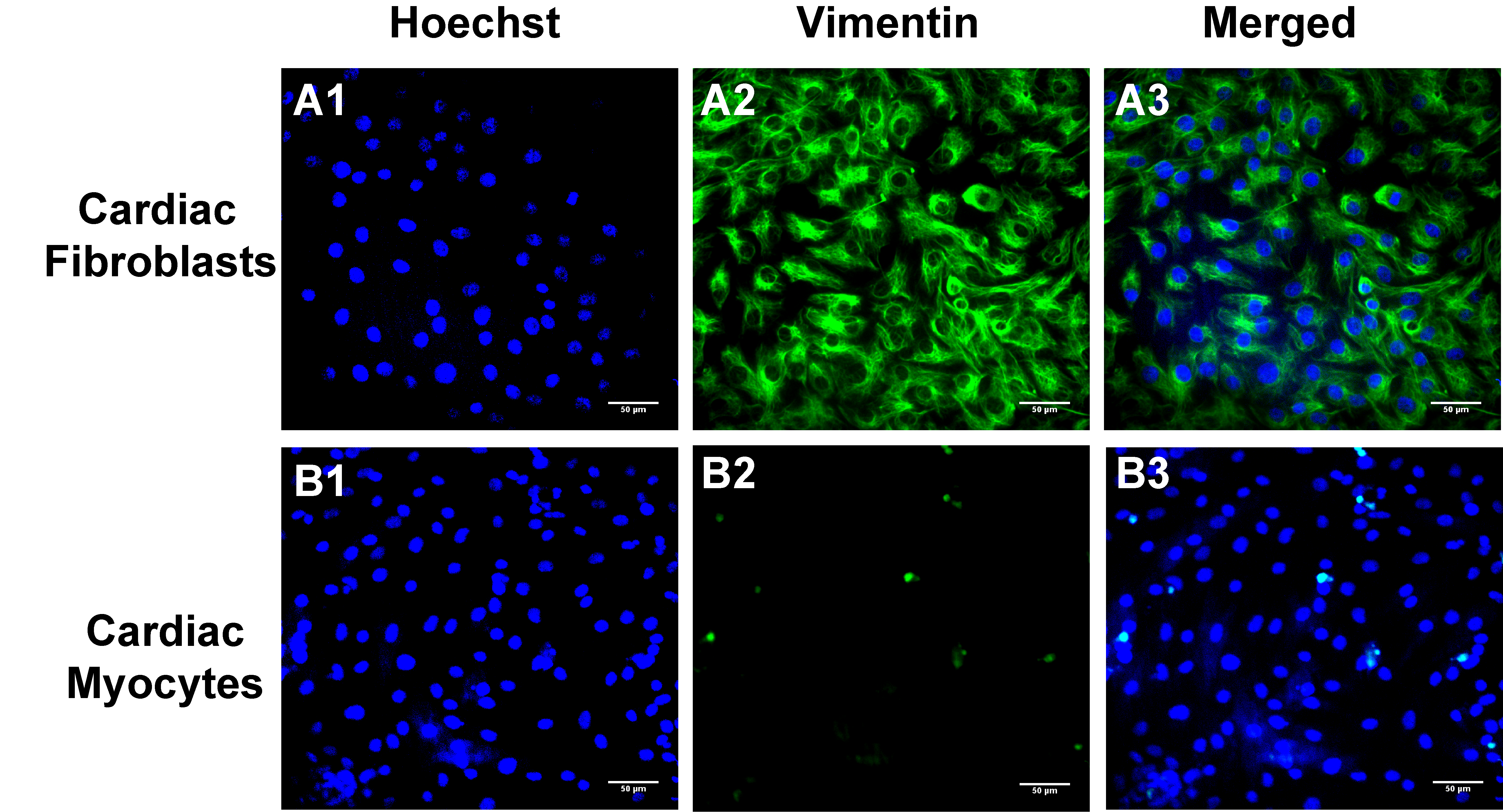

Supplement: Supplemental Figure 1 — Vimentin immunofluorescence microscopy. Immunostaining for vimentin demonstrates no fibroblast contamination of neonatal rat cardiac myocyte cultures. (A1–A3) Representative microscopy images of a neonatal rat cardiac fibroblast culture shows co-localization of Hoechst and vimentin-labeling. (B1–B3) Representative microscopy images of a neonatal rat cardiac myocyte culture shows nuclear labeling with Hoechst (blue), but no vimentin-positive cells. [file Image_1.TIF]

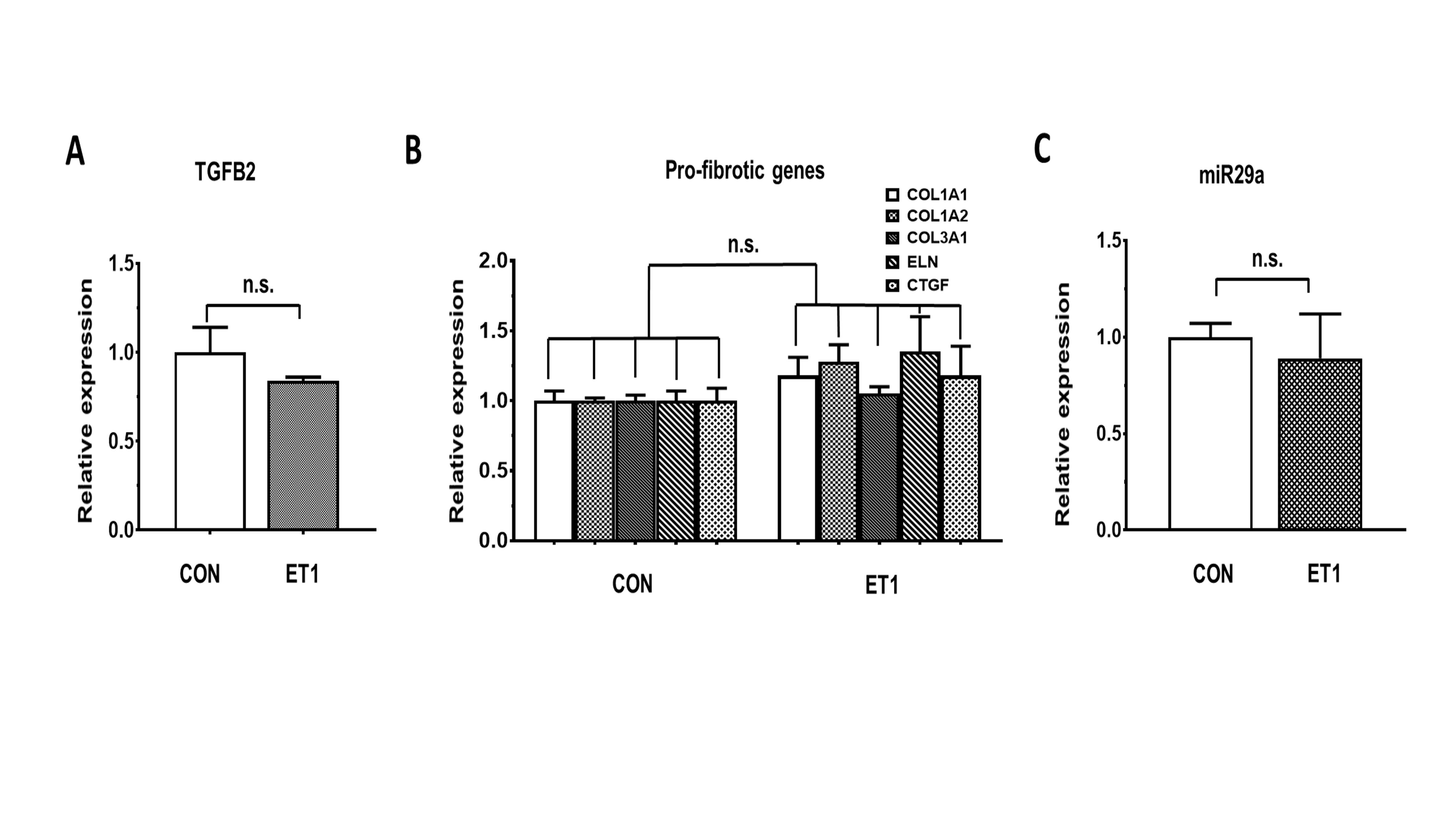

Supplement: Supplemental Figure 2 — ET1 has no effect on miR-29a and pro-fibrotic gene expression in cultured neonatal rat cardiac fibroblasts. (A–C) Summary of gene expression of TGFB2, CTGF, collagen genes, and miR-29a show no difference in expression between untreated (control) and fibroblasts treated with ET1 (100 nM for 24 h) [CON, control; ET1, endothelin1 (100 nM) treatment for 24 h. Data are expressed as mean ± S.D; n = 3 (biological replicates); all experiments were repeated three times. Two tailed Student's t-test was used to determine significance; n.s., non-significant]. [file Image_2.TIF]

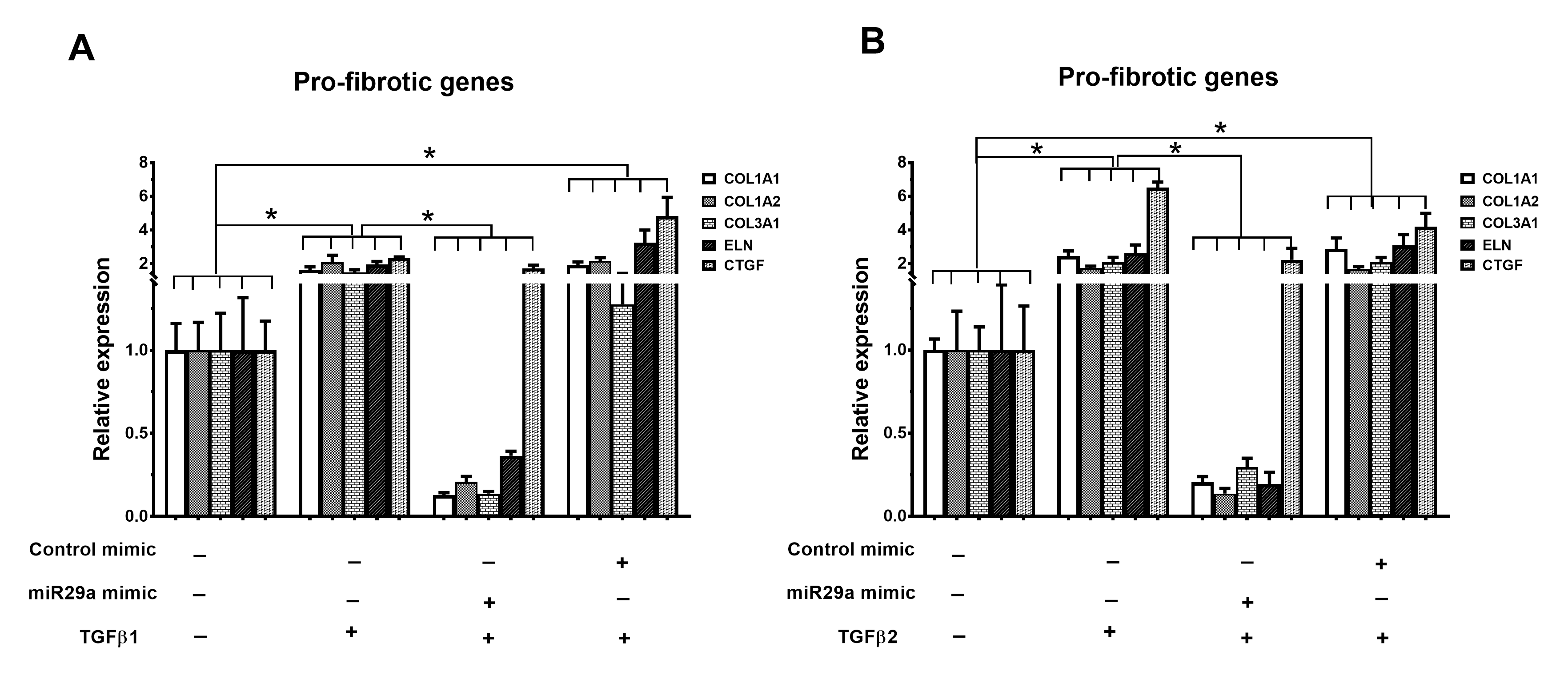

Supplement: Supplemental Figure 3 — TGβ1/2 stimulated collagen expression in neonatal rat cardiac fibroblast cultures is antagonized by miR-29-mimic. Cardiac fibroblasts were either treated with 5 nM concentration of either miR-29a-mimic or control-mimic for 6 h, prior to stimulation with TGFβ1 or TGFβ2 for 24 h. (A) miR-29a-mimic suppressed TGFβ1-stimulated expression of profibrotic miR-29 targets, but not CTGF, which is not a miR-29 target gene (linear fold change is presented). (B) miR-29a-mimic suppressed TGFβ2-stimulated expression of profibrotic miR-29 targets, but not CTGF (linear fold change is presented) [data are expressed as mean ± S.D.; n = 3 (biological replicates) for each condition; all experiments were repeated three times. One-way ANOVA and Tukey's test were used to compare treatment conditions; *P < 0.05]. [file Image_3.TIF]

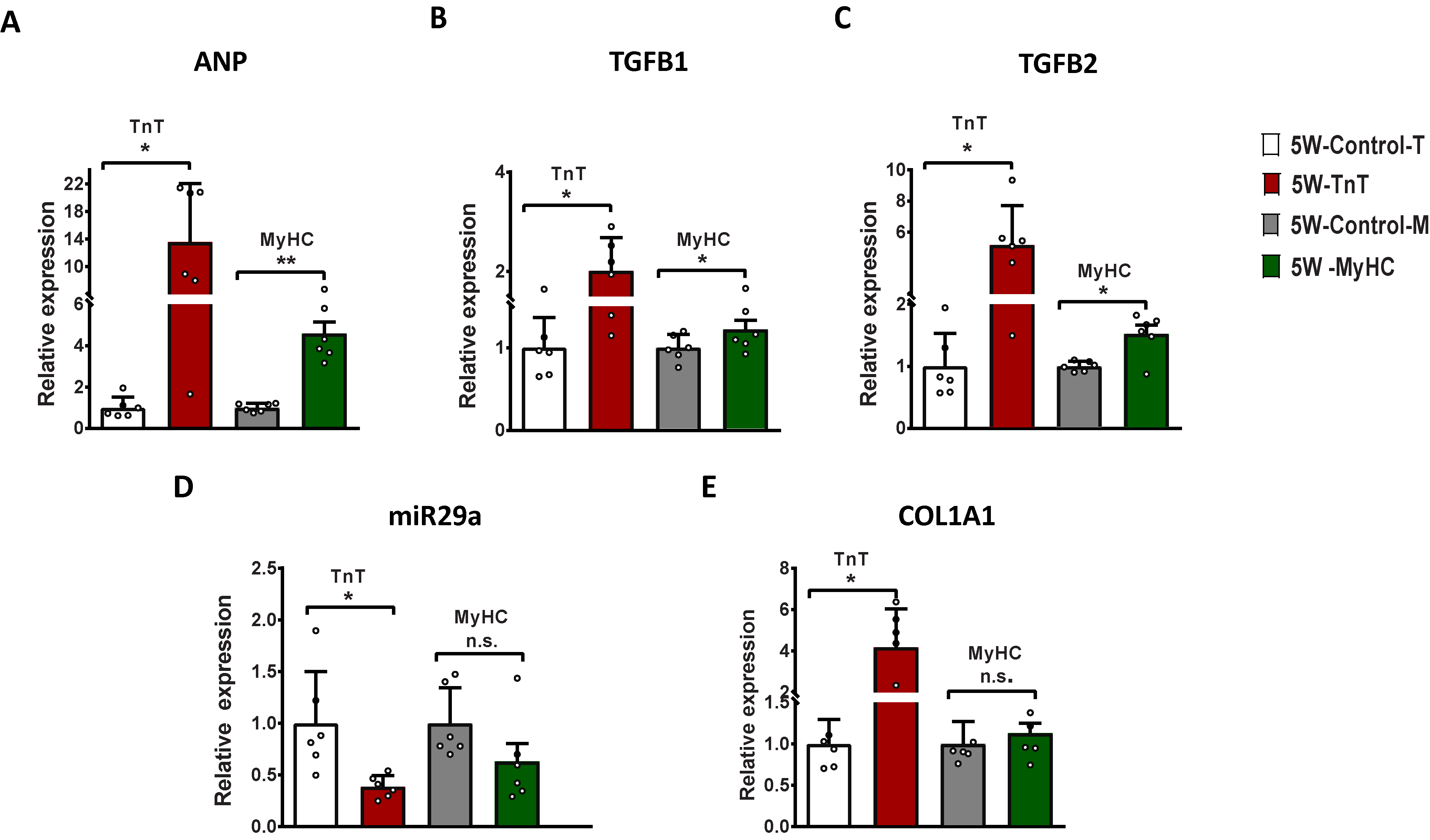

Supplement: Supplemental Figure 4 — Allele-specific differences in expression of miR-29a, ANP, TGFB1/2, COL1A1 in LV of female HCM mice/littermate controls at 5 weeks of age. (A–E) Allele-specific differences in expression of ANP, TGFB1/2, miR-29a, and COL1A1 in female TnT and MyHC mutant mouse -LV and littermate control-LV at 5 weeks of age (Control-T, TnT-littermate control; TnT, TnT-mutant; Control-M, MyHC-littermate control; MyHC, MyHC-mutant) (data are expressed as mean ± S.D.; n = 6 in each group. The two-tailed unpaired Student's t-test was used to compare TnT mutants and MyHC mutants with respective littermate controls; *P < 0.05; n.s., non-significant). [file Image_4.TIF]

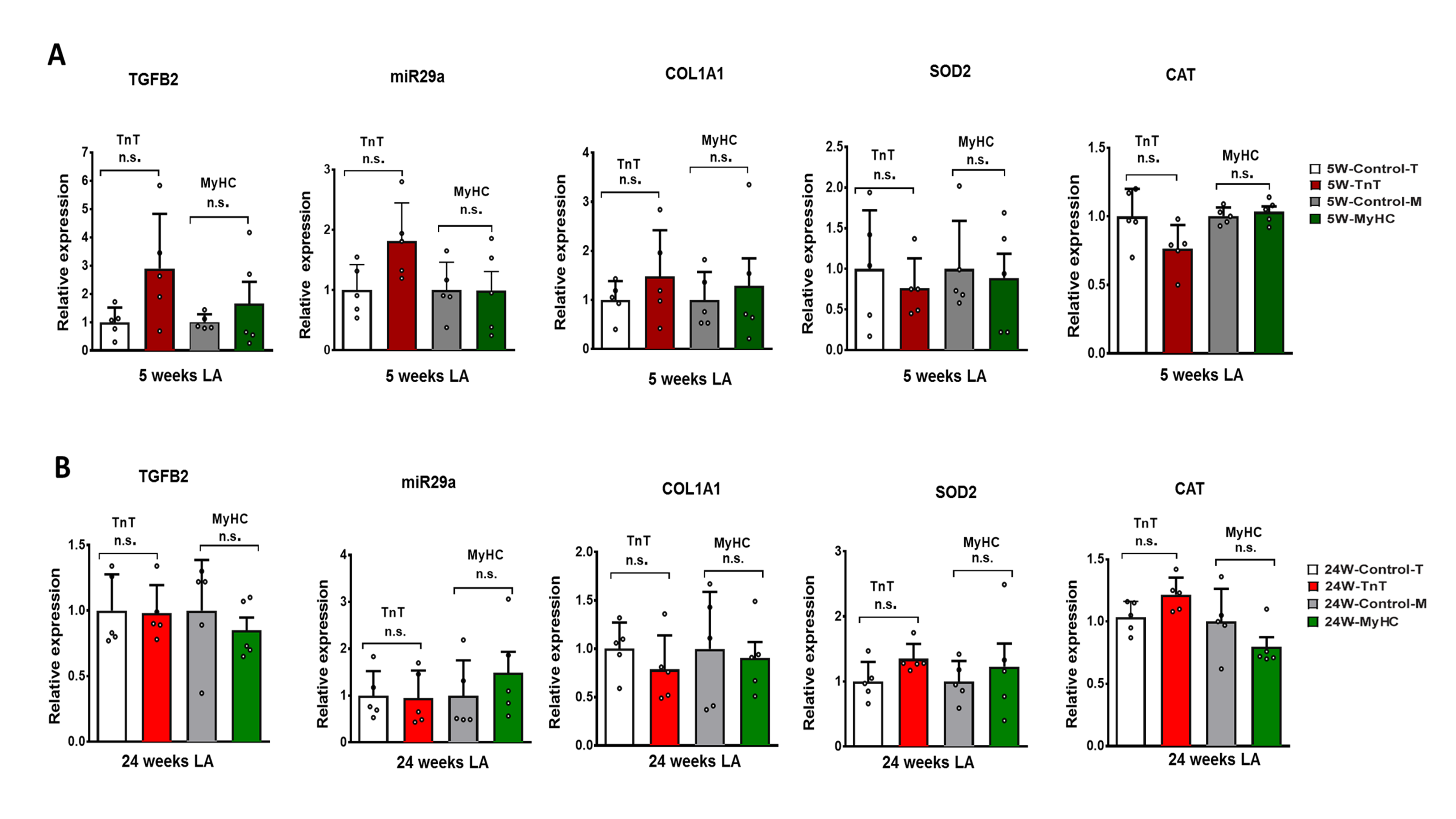

Supplement: Supplemental Figure 5 — Gene expression in left atrial appendage of HCM mice and littermate controls at 5 and 24 weeks of age. (A,B) No difference in expression of miR-29a/b/c, select pro-fibrotic and redox genes between left atrial appendage of mutant mice and littermate controls at 5 and 24 weeks of age (control-T, TnT-littermate control; TnT, TnT-mutant; Control-M, MyHC-littermate control; MyHC, MyHC-mutant) (data are expressed as mean ± S.D.; n = 5 for each group. The two-tailed unpaired Student's t-test was used to compare TnT mutants and MyHC mutants with respective littermate controls; n.s., non-significant). [file Image_5.TIF]
